# Effect of Ethyl Acetate on the Defatting of Leaves in the Extraction of *Stevia rebaudiana* Bertoni

**DOI:** 10.17113/ftb.62.03.24.8492

**Published:** 2024-09

**Authors:** Liliana Celaya, Nicolás Kolb Koslobsky

**Affiliations:** 1 Department of Chemical Engineering, National Scientific and Technical Research Council (CONICET UNaM), Félix de Azara 1552, 3300-Posadas, Argentina; 2 Central Laboratory, School of Exact, Chemical and Life Sciences, Misiones National University, Félix de Azara 1552, 3300-Posadas, Argentina

**Keywords:** production of stevioside and rebaudioside A, * Stevia rebaudiana* leaves, percolation bed, pre-extraction with ethyl acetate

## Abstract

**Research background:**

The process for producing purified steviol glycosides from *Stevia rebaudiana* leaves (stevia) generally involves pretreatments, extraction, purification and crystallization. Pre-extraction or defatting can sometimes be a part of this process. It can remove impurities of low polarity, such as chlorophyll and fatty compounds. Nonpolar solvents can be used to defat stevia leaves.

**Experimental approach:**

In this study, we investigated ethyl acetate as a pre-extraction solvent for the defatting of dried and crushed stevia leaves. We compared pure ethyl acetate and water-saturated ethyl acetate as pre-extraction solvents in percolation extraction. We then evaluated the effects of pre-extraction on the concentration and purity of the extracts obtained with ethanol/water solvents.

**Results and conclusions:**

The recovery of nonpolar solvents was 2.3–3.9 % in pure ethyl acetate and 3.4–4.5 % in water-saturated ethyl acetate (from 40 to 60 °C). A low steviol glycoside loss can occur only with water-saturated ethyl acetate (on dry mass basis <0.5 %). In the Soxhlet extraction, the obtained yields were 8.43 with pure ethyl acetate and 10.44 % with water-saturated ethyl acetate. The steviol glycoside loss in the Soxhlet extraction was 10.70 % with water-saturated ethyl acetate. Defatted and non-defatted leaves were extracted with two ethanol/water solvents. Comparison of the results showed higher concentrations of glycoside in the pretreated leaves.

**Novelty and scientific contribution:**

The pre-extraction with ethyl acetate followed by the extraction with ethanol/water solvent lead to a higher concentration of steviol glycosides and a higher purity of the extracts. Ethyl acetate can be used as a pre-extraction solvent for the defatting of stevia leaves in the industrial production of this sweetener.

## INTRODUCTION

*Stevia rebaudiana* (Bert) Bertoni, commonly called stevia, is a rich source of natural sweeteners steviol glycosides. These organic compounds are chemically composed of glycoside groups linked to the diterpene steviol. In 2007, the Joint FAO/WHO Expert Committee on Food Additives approved 10 steviol glycosides as additives (*1*). Recently, 20 new steviol glycosides have been isolated and characterised (*2*). Steviol glycosides are used in many countries in food formulations and for different pharmaceutical purposes (*3*).

The percentage of steviol glycosides in stevia leaves varies between 4 and 20 %. The main steviol glycosides in the leaves are stevioside (1–10 %), rebaudioside A (2–13 %) and rebaudioside C (0.5–1.5 %). Other steviol glycosides are usually present in smaller amounts (*4*-*6*). Stevioside (Stv) and rebaudioside A (RbA) are largely responsible for the sweetness of stevia leaves, extracts and commercial products (*7*, *8*). RbA, which has an additional glucose unit relative to Stv, is superior in terms of taste quality (*9*). Rebaudioside C (RbC), with different sugar moiety (rhamnose) compared to RbA, has been shown to act as a flavour enhancer together with RbA. Rebaudioside D (RbD), which has desirable sweetening properties, occurs naturally in stevia plants in low concentrations (*10*). The physicochemical properties of steviol glycosides differ from each other.

Steviol glycoside production generally involves pretreatment, extraction, purification and crystallization. The first step is a solid-liquid extraction of the pretreated stevia leaves (*11*, *12*). The steviol glycosides are extracted from the leaves with water or alcohol (*13*, *14*). The product of the extraction step is a coloured and dense mixture containing steviol glycosides along with other components, such as saccharides, proteins, oils, pigments, phenolic compounds, gums, colloids and other impurities (*15*, *16*). The obtained extract is usually subjected to a multi-step purification to obtain the individual components, namely Stv, RbA, RbC and mixtures of steviol glycosides (*13*, *17*, *18*).

Pre-extraction or defatting can also be used in this process to remove impurities of low polarity, for example chlorophyll and fatty compounds, by boiling the plant material with the solvent (*19*-*21*). Nonpolar solvents such as hexane, petroleum ether or CO_2_ can be used (*22*-*24*). In this context, there are some studies investigating alternatives to carry out this separation step with different procedures and solvents (*25*, *26*).

Previously, Formigoni *et al*. (*25*) used a column to treat stevia leaves with ethanolic solvent. Ciotta *et al*. (*26*) used leaves pretreated with ethanol for the percolation extraction of steviol glycosides with boiling water as solvent. In a previous work, we optimized the temperature and percentage of ethanol to extract Stv and RbA from stevia leaves in a percolation extractor. The optimal extraction conditions that gave the best steviol glycoside extraction kinetics were 70 °C and 35 % ethanol (*14*). In addition, the optimal extraction conditions that gave maximum purity were 70 % ethanol at 30–70 °C. In the present study, we aim to investigate the effects of using ethyl acetate to defat dried and crushed stevia leaves. We compared pure ethyl acetate and water-saturated ethyl acetate as solvents for pre-extraction, and investigated the effect of pre-extraction on the concentration and purity of stevia extracts obtained with ethanol/water as solvent.

## MATERIALS AND METHODS

### Chemicals

Ethanol and ethyl acetate were obtained from Cicarelli (Reagents S.A., San Lorenzo, Argentina). The distilled water used for the extraction was deionized in a Milli-Q system (Millipore Sigma, Bedford, MA, USA). HPLC-grade acetic acid, acetonitrile, water and ethanol were obtained from Merck (Darmstadt, Germany). Crystals of stevioside (Stv; 98.5 % purity), rebaudioside A (RbA; 99.5 % purity), rebaudioside B (RbB; 99.5 % purity) and rebaudioside C (RbC; 99.5 % purity) were obtained in-house (Project 16Q1204-IDP) by preparative column chromatography and successive recrystalizations (*27*). The crystals were compared with steviol glycoside United States Pharmacopeia (USP) reference standard solution (Sigma–Aldrich, Merck, Steinheim, Germany). Other chemicals used were of analytical grade.

Binary solvents were prepared at room temperature. The binary solvent used for defatting of leaves (water-saturated ethyl acetate) was prepared by adding water to ethyl acetate up to saturation of 3.3 % *m*/*m* (*28*). The binary solvents (ethanol/water solvents) used for the extractions were prepared according to Celaya *et al.* (*3*).

### Raw materials

Stevia (*Stevia rebaudiana*) was harvested at the experimental farm in Posadas (Departamento Capital, Argentina) when the flowers were opened at 0–5 %. The samples were collected after sun-drying for 3–4 days. The dried leaves were crushed to obtain particles with mesh size of 5–40. The residual moisture content was separated from the leaves by drying the samples at (60±2) °C until they reached a constant mass.

### Pre-extractions

Pure ethyl acetate (PEtAc) and water-saturated ethyl acetate (SEtAc) were used to defat stevia leaves. Ground stevia leaves used for the assays were dried at 60 °C until the remaining water was removed.

The solid/liquid pre-extractions were carried out using a laboratory scale percolator according to Celaya *et al*. (*14*). The percolation temperature was controlled by a thermostatic system with recirculation of heating water (model CT1150; Schott Geräte, Mainz, Germany). The extraction temperature was measured with three thermometers.

For each experiment, 200 g of pre-dried leaves were packed and impregnated with 1300 mL of solvent. After impregnation (10 min), the solvent was eluted by gravity and 5 fractions of 200 mL were obtained. The total percolation time was 60 min; the mean flow rate was (16.7±1.4) mL/min. After extraction, the 5 fractions were rapidly cooled to room temperature and mixed. Each extraction was carried out in duplicate. Total volume of extracts (1000 mL) was used to compare the results of different working conditions. The extracts were filtered and diluted in *m*(ethanol)/*m*(water)=70:30 *m*/*m* for analysis.

### Extractions with ethanol/water mixtures

The mixed solvent was removed by evaporation at 60 °C. The samples were then vacuum dried to constant mass. Defatted stevia leaves were dried at 60 °C for three days and then they were vacuum dried until the remaining solvent was removed. Untreated stevia leaves were dried at 60 °C after the extractions.

Defatted and untreated stevia leaves were extracted at 70 °C as previously described (*14*). Two ethanol/water solvents were used: *w*(ethanol)=35 % (EtOH35) and 70 % (EtOH70). During percolation, the binary solvent was eluted by gravity and 4 fractions of 250 mL were obtained. The total percolation time was 60 min. The total extract volume (1000 mL) was used to compare the results of different operating conditions.

### Soxhlet extraction

For comparison, Soxhlet extractions were carried out using PEtAc and SEtAc as defatting solvents. Ground stevia leaves were pre-dried at 60 °C for 2 h and then 20 g were placed in the extraction thimble (*14*). The thimble was placed in the Soxhlet extractor. The plant material was extracted with 500 mL of solvent for 8.5 h. The remaining solvent was removed by evaporation at 60 °C until constant mass was reached. The extracted materials and extracts were used for analysis. Soxhlet extractions were conducted in duplicate.

### Analytical determinations

The obtained extracts were filtered and diluted to *w*(ethanol)=70 % for analysis. The steviol glycosides in leaves and extracts were quantified with a high-performance liquid chromatography diode array detector (HPLC-DAD) using the external standard method (*29*). Stock solutions of 0.2–1.0 g/L of the standard compounds (Stv, RbA and RbC) in *w*(ethanol)=70 % were prepared for the calibration curve. The compounds in each sample were identified by comparing their retention times with those of the standards. Rebaudioside D (RbD) was quantified in the same way as RbA.

Ground stevia leaves contained the following mass fractions on dry mass basis of individual steviol glycosides (in %): RbA (10.3±0.4), Stv (3.0±0.1), RbC (1.1±0.1), RbB (0.4±0.1) and RbD (0.4±0.1). The mass fraction of all steviol glycosides was (15.2±0.4) %. The moisture mass fraction was (5.3±0.5) %.

The extraction process was monitored by measuring the mass fractions of Stv, RbA and RbC in leaves and extracts. The process variables determined were: concentration of glycosides *γ*/(g/L) and their recovery and purity expressed as mass fraction (*w*/%) on dry mass basis (*14*). The total extracted solid (TES) mass fraction was measured after drying 10 mL of extract at 80–102 °C on a tared steel plate until a constant mass (*27*).

### Statistical analyses

Statistical analyses were performed with R Studio, v. 4.0.3 (*30*). Mean values were compared using a two-way ANOVA and *post-hoc* Tukey’s tests to determine differences with statistical significance. Differences were considered significant at p<0.05.

## RESULTS AND DISCUSSION

### Effect of defatting with ethyl acetate

**Fig. 1:**
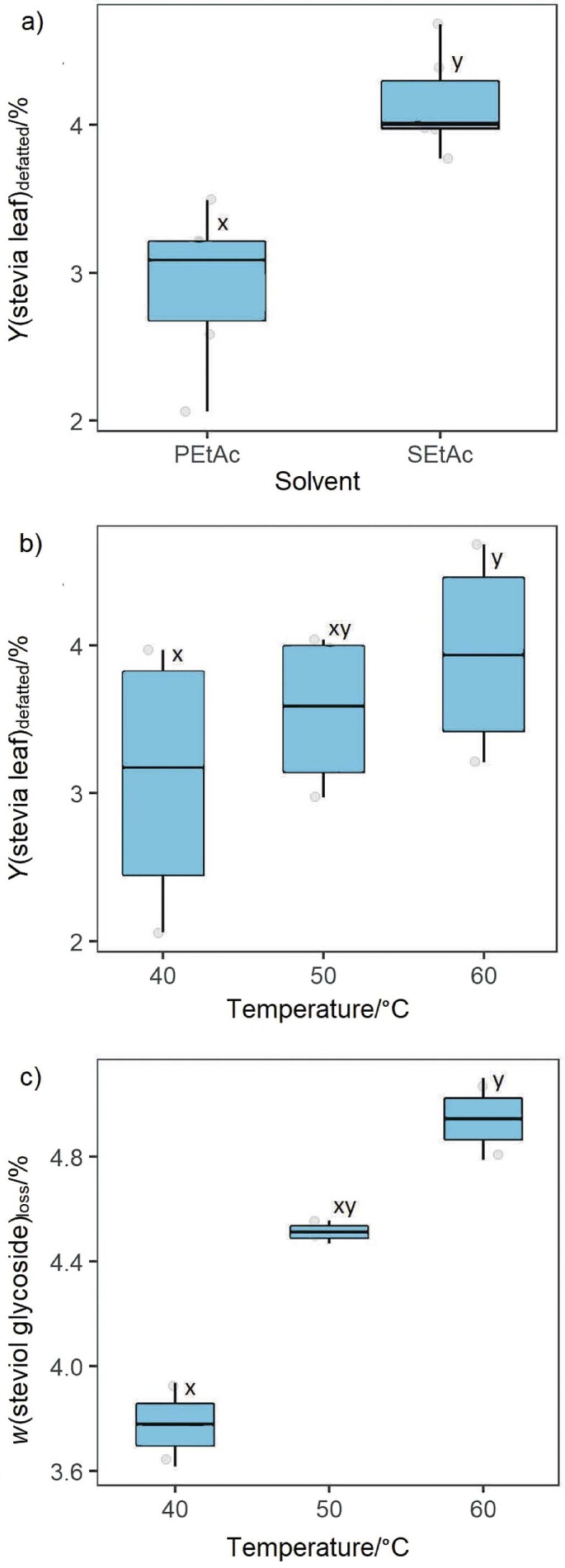
Dependence of stevia leaf defatting yield on dry mass basis on: a) defatting solvent (p=0.00381) and b) temperature (p<0.00000) and c) steviol glycoside loss, in the pre-extraction with SEtAc (p=0.0423). x and y mark mean values that are significantly different (p<0.05). PEtAc=pure ethyl acetate, SEtAc=water-saturated ethyl acetate

Research into the defatting step would be of interest for the industrial stevia process. To investigate the potential of defatting process with ethyl acetate, two nonpolar solvents, PEtAc and SEtAc, were tested. Fig. 1 shows the recovery of nonpolar compounds with PEtAc and SEtAc at different temperatures (40, 50 and 60 °C). The defatting of stevia leaves shows a dependence on solvent (Fig. 1a) and temperature (Fig. 1b). There was no interaction between solvent and temperature (p=0.19049). The recovery of steviol glycosides on dry mass basis in the pre-extraction with PEtAc varied from 2.3 to 3.9 % (from 40 to 60 °C), while with SEtAc, it varied from 3.4 to 4.5 %.

Various pretreatment strategies have been used in the stevia industry to increase the efficiency of the extraction (*15*). Pretreatments of leaves may be appropriate depending on their lipid content (*22*-*24*). Nonpolar solvents such as hexane or petroleum ether can be used. In our study, we investigated ethyl acetate as a pre-extraction solvent for the defatting of dried and crushed stevia leaves. To the best of our knowledge, there are no reports on the use of ethyl acetate in the pre-extraction of steviol glycosides from stevia leaves. Our results show that ethyl acetate and water-saturated ethyl acetate have great potential for industrial use as a pre-extraction solvent for defatting stevia leaves.

Multiple extractions were carried out in a Soxhlet apparatus with PEtAc and SEtAc as defatting solvents. The obtained yields on dry mass basis were (8.4±1.6) and (10.4±0.9) of total solids, with PEtAc and SEtAc, respectively. These analytical results show a great potential of the nonpolar solvent for the extraction of compounds other than steviol glycosides from stevia leaves.

Previous work has investigated the performance of different solvents in the defatting of stevia leaves. According to these results, oil recovery in petroleum ether extraction was 3.7–4.3 % of the leaves (*22*, *31*). In cold extraction with petroleum ether (*t*_b_=40–60 °C), the oil recovery was 3.15–5.45 % (*19*), and in Soxhlet extraction 2.00–6.00 %. In the present study, the best condition for defatting the leaves was 60 °C when using SEtAc (Fig. 1).

The polarity indices of the solvents can partly explain the observed behavior. The normalized polarity indices *E*_T_ range from 0.000 for the least polar solvent (tetramethylsilane) to 1.000 for the most polar solvent (water). The normalized *E*_T_ values are <0.125 for petroleum ether, 0.009 for hexane and 0.228 for ethyl acetate (*32*, *33*). The low polarity of ethyl acetate could be partly responsible for the nonpolar recovery in the purification of stevia leaves.

In some purification processes, ethyl acetate can be used industrially as a replacement for petroleum ether (*32*). To achieve complete leaching of the plant material, additional solvents need to be added to the percolator bed (*14*). Additional volumes of solvent are required in pre-extractions with the nonpolar solvent.

### The loss of steviol glycosides during pre-extraction

During the pretreatment of leaves, several substances can be extracted together with nonpolar impurities. These co-extracted compounds may be steviol glycosides. The loss of steviol glycosides during pre-extraction was monitored in the defatting experiments. The loss of steviol glycosides was not observed during the pre-extractions with PEtAc. The graphical results of steviol glycoside loss in the pre-extractions with SEtAc are shown in Fig. 1c. Water-saturated ethyl acetate SEtAc resulted in a loss of 3.9–4.6 % of steviol glycosides (from 40 to 60 °C).

Previously, Formigoni *et al*. (*25*) used ethanolic solvent for the pretreatment of stevia leaves and found a decrease in fatty acid content (68 %) and a loss of sweeteners of 9.5 % (mainly RbA). In the present study, the loss of steviol glycosides at 60 °C with SEtAc was 4.6 % (mainly RbA and Stv).

The multiple extractions carried out with PEtAc yielded 8.4 % with a loss of steviol glycosides of less than 1.0 %. In the Soxhlet extraction with PEtAc, mainly Stv and RbA with nonpolar impurities were extracted. Multiple extractions with SEtAc yielded 10.44 % with a loss of steviol glycosides of (10.7±6.2) %. An important feature of the use of SEtAc in Soxhlet extraction is that the greatest loss occurs in Stv (40.8±5.2) compared to RbA (3.9±2.9) and RbC (16.1±5.4). The resulting defatted leaves have a mean ratio of *w*(RbA)/*w*(Stv)=4.9.

### Extraction with ethanol/water solvents

Defatted and non-defatted leaves were extracted with two ethanol/water solvents: EtOH35 and EtOH70. Table 1 shows the concentrations of steviol glycosides in the raw materials used for the ethanol/water extractions. Table 2 shows the results of the extractions with binary solvents. These preliminary results show that the concentrations of steviol glycosides are higher in the pretreated leaves.

**Table 1:** Mass fraction on dry mass basis of main steviol glycosides present in the raw materials used for the ethanol/water extractions

	*w*(steviol glycoside)/%		
Raw material	Stv	RbA	RbC
Untreated	(2.90±0.07)^a^	(10.22±0.06)^a^	(1.05±0.02)^a^
PEtAc	(3.34±0.01)^b^	(11.05±0.04)^b^	(1.11±0.03)^a^
SEtAc	(3.30±0.01)^b^	(10.66±0.12)^ab^	(1.02±0.02)^a^

The values with different letters in superscript in the same column are significantly different (p<0.05). Stv=stevioside, RbA=rebaudioside A, RbC=rebaudioside C, PEtAc=pure ethyl acetate and SEtAc=water-saturated ethyl acetate

**Table 2:** Concentration of steviol glycosides and TES during extraction of untreated and pretreated stevia leaves

		*γ/*(g/L)				
Pre-extraction	Solvent	Stv	RbA	RbC	TSG	TES
Untreated	EtOH35	(3.9±0.2)^a^	(15.610.4)^a^	(1.9±0.1)^ab^	(21.84±0.02)^a^	(90.7±0.7)^a^
	EtOH70	(3.8±0.2)^a^	(15.98±0.03)^a^	(1.43±0.01)^a^	(21.2±0.2)^a^	(70.1±1.5)^b^
PEtAc	EtOH35	(6.13)^b^	(19.80)^b^	(2.14)^b^	(29.27)^b^	(89.71)^a^
	EtOH70	(5.35)^b^	(17.49)^b^	(1.82)^a^	(25.73)^b^	(77.62)^b^
SEtAc	EtOH35	(6.10)^b^	(20.68)^b^	(2.13)^b^	(30.07)^b^	(86.88)^a^
	EtOH70	(5.56)^b^	(19.03)^b^	(1.88)^ab^	(27.70)^b^	(76.87)^b^
p*	pre-extraction	0.000921	0.01150	0.01421	0.00411	>0.05
	solvent	>0.05	>0.05	0.00466	>0.05	0.0032
	pre-extraction × solvent	>0.05	>0.05	>0.05	>0.05	>0.05

*Statistical significance for linear models in R Studio (*30*). The results were obtained in a laboratory scale percolator. Mean values with different letters in superscript in the same column are significantly different (p<0.05). EtOH35=*w*(ethanol)=35 %, EtOH70=*w*(ethanol)=70 %, Stv=stevioside, RbA=rebaudioside A, RbC=rebaudioside C, TSG=total steviol glycosides, TES=total extracted solids, PEtAc=pure ethyl acetate and SEtAc=water-saturated ethyl acetate

The effect of the extraction solvents on the purity is shown in Table 3. The comparison of the results shows that a pre-extraction with ethyl acetate followed by an extraction with ethanol/water leads to a higher purity of the extracts. According to our earlier studies, EtOH70 (at 70 °C) is the optimum extraction condition that gives maximum purity (*14*).

**Table 3:** Purity of ethanol/water extracts

		*w*/%				
Pre-extraction	Solvent	Stv	RbA	RbC	TSG	*w*(RbA)/*w*(Stv)
Untreated	EtOH35	(4.1±0.2)^a^	(17.2±0.6)^a^	(2.1±0.2)^a^	(23.48±0.02)^a^	(4.05±0.09)^a^
	EtOH70	(4.1±0.2)^a^	(22.8±0.5)^ab^	(2.05±0.02)^a^	(30.3±0.9)^ab^	(4.2±0.2)^a^
PEtAc	EtOH35	(6.83)^b^	(22.07)^ab^	(2.39)^b^	(32.63^ab^	(3.23)^b^
	EtOH70	(6.89)^b^	(22.53)^ab^	(2.34)^b^	(33.15)^ab^	(3.27)^b^
SEtAc	EtOH35	(7.02)^b^	(23.80)^ab^	(2.45)^b^	(34.61)^ab^	(3.39)^b^
	EtOH70	(7.23)^b^	(24.76)^b^	(2.45)^b^	(36.03)^b^	(3.42)^b^
p*	pre-extraction	0.000921	0.00229	0.00937	0.00227	0.0011
	solvent	>0.05	0.00156	>0.05	0.00297	>0.05
	pre-extraction×solvent	>0.05	0.0512	>0.05	0.0937	>0.05

*Statistical significance for linear models in R Studio (*30*). For TSG and RbA the model used includes the interaction of pre-extraction and solvent. Mean values with different letters in superscript in the same column are significantly different (p<0.05). EtOH35=*w*(ethanol)=35 %, EtOH70=*w*(ethanol)=70 %, Stv=stevioside, RbA=rebaudioside A, RbC=rebaudioside C, TSG=total steviol glycosides, PEtAc=pure ethyl acetate and SEtAc=water-saturated ethyl acetate

When comparing the purity results of percolation with ethanol/water solvents, we found that SEtAc is the best solvent for obtaining an extract with the best purity (Table 3). The results show purity values of 34.6 and 36.0 % with EtOH35 and EtOH70, respectively.

In addition to the steviol glycoside loss in the pre-extraction with SEtAc, a lower *w*(RbA)/*w*(Stv) ratio was measured with ethyl acetate than with untreated leaves (Table 3). Without pre-extraction, several low-polarity impurities can be co-extracted in the extraction process. Pre-extraction promotes a higher process yield and better purity. However, co-extracted substances, including steviol glycosides, can also be extracted together with nonpolar impurities. In addition, the extraction of steviol glycosides with polar solvents requires subsequent removal of solvent residues (*20*).

### Effect of pre-extraction on the efficiency of extraction with ethanol/water solvents

**Fig. 2:**
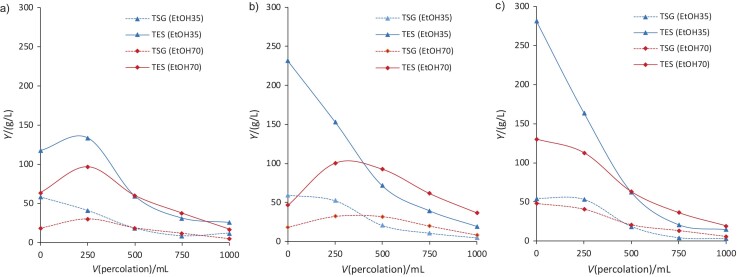
Extraction yield (*Y*) of steviol glycosides and total extracted s with different solvents *w*(ethanol)=35 % (EtOH35) and 70 % (EtOH70) (percolation values): a) untreated leaves, b) leaves pretreated with PEtAc, and c) leaves pretreated with SEtAc. TSG=total steviol glycosides, TES=total extracted solids, PEtAc=pure ethyl acetate and SEtAc=water-saturated ethyl acetate

The efficiency of the extraction of steviol glycosides and total extracted solids (TES) with EtOH35 and EtOH70 was monitored in the percolation extractions. Percolation concentrations of steviol glycosides and TES ​​were measured. Fig. 2 shows the percolation data plotted for untreated leaves (Fig. 2a), leaves pretreated with PEtAc (Fig. 2b) and leaves pretreated with SEtAc (Fig. 2c). According to the obtained results, EtOH35 is superior to EtOH70, as it allows a faster recovery of steviol glycosides (*14*). In the percolation process, a large part of the TES appeared immediately in the pretreated leaves (Fig. 2b and Fig. 2c). Strikingly, the use of SEtAc significantly increased the recovery of steviol glycosides in the percolation extraction. This result adds to the higher purity achieved when using EtAc.

Finally, a critical consideration must be made to define the application of a defatting operation in the process. This operation requires an additional step in the extraction process, which can increase costs and time, and require the use of complex equipment (*15*). For an environmentally friendly process, the possible use of ethyl acetate extracts as a source of chlorophyll and fatty compounds can be considered in future studies, as well as the possible recycling of ethyl acetate in the stevia process. A solvent recycling process can be used for the recovery and subsequent reuse of ethyl acetate, which is environmentally friendly from an ecological point of view.

## CONCLUSIONS

Defatting can be suitable for the pretreatment of stevia leaves in industrial processes. In this study, we report about the use of ethyl acetate for the defatting of stevia leaves in a percolator extractor. We investigated pure ethyl acetate (PEtAc) and water-saturated ethyl acetate (SEtAc) and the results show that ethyl acetate is appropriate for the removal of nonpolar impurities from stevia leaves. Pre-extraction with ethyl acetate promotes a higher extraction yield of steviol glycosides and their highest purity. SEtAc removes impurities from stevia leaves more efficiently, but it may cause a loss of steviol glycosides.

Ethyl acetate can be used as a pre-extraction solvent for defatting stevia leaves. Moreover, the solvent recycling operation can make the process economic and environmentally friendly. In this regard further investigation of the pre-extraction with ethyl acetate and its effect on stevia purification should be carried out in the future.
